# Short term fluctuating temperature alleviates *Daphnia* stoichiometric constraints

**DOI:** 10.1038/s41598-021-91959-w

**Published:** 2021-06-11

**Authors:** Esteban Balseiro, Cecilia Laspoumaderes, Facundo Smufer, Laura Wolinski, Beatriz Modenutti

**Affiliations:** 1 Laboratorio de Limnología, INIBIOMA, CONICET-University of Comahue, Quintral 1250, 8400 Bariloche, Argentina; 2 Alfred-Wegener-Institut Helmholtz-Zentrum Für Polar- und Meeresforschung, Helgoland, Germany; 3 Groupe de Recherche Interuniversitaire en Limnologie, (GRIL), Département des Sciences Biologiques, Université du Québec À Montréal, Montréal, QC Canada; 4 Laboratorio de Ecología, Fisiología y Evolución de organismos acuáticos, CADIC, CONICET, Ushuaia, Argentina

**Keywords:** Ecophysiology, Freshwater ecology, Ecology

## Abstract

In this study, we analysed how short term temperature fluctuation interacts with nutrient limitation in the vertical migrating *Daphnia commutata*. We hypothesize that short term (daily) temperature fluctuation will alleviate nutrient limitation. We carried out experiments analysing growth rates, phosphorus and RNA content of *D. commutate* grown under four different temperature regimes and two P-limited conditions. Our experiments showed that individuals grown under fluctuating temperature grew more than at the mean temperature. We estimated the expected sizes for the 15 °C treatment based on the Q_10_ and for the fluctuating temperature treatment. These expected sizes for both treatments resulted well below the observed ones. The P and RNA content of individuals grown at 10 °C were significantly higher than those at 20 °C, and when individuals grown at 10 °C were translocated to 20 °C they exerted an increased growth rate. Our results suggest that, under a regime of diel vertical migration, the temperature alternation would allow migrating organisms to alleviate the effect of severe nutrient limitation maintaining population growth. Under a scenario of global warming, where epilimnetic temperatures will increase, lake temperature will interact with nutrient limitation for consumers, but, organisms may be able to face these changes if they can still regularly move from a cold hypolimnion to a warmer epilimnion.

## Introduction

In ectotherms, temperature governs most physiological processes, hence, even small changes in environmental temperature may lead to exponential changes in their metabolism and growth^1,2^. In addition, the growth rate hypothesis (GRH) states that growth in invertebrates is positively related to somatic phosphorus (P) content due to the increased demand for ribosomal RNA production needed to sustain rapid growth^3–5^. As a consequence, P limitation lowers actual growth rates^6^. However, when different temperatures were considered in a temperature gradient field work, the observed growth rates of invertebrates did not reflect the GRH predicted P-RNA-growth rate relationship^7,8^.

Most experimental evidence show that phosphorus requirements of zooplankton are affected by temperature^9–14^. However, whether organisms need more or less P at different temperatures is still an open question^15^, that has brought some controversy in how these two factors interact. In *Daphnia magna*, low temperature increases alkaline phosphatase in other to compensate for the lower specific enzyme activity^10^. In this sense, there is evidence that organisms P content can differ between organisms living at different temperatures with cold-acclimated poikilotherms having higher P contents than warm-exposed conspecifics^4,16^. The concomitant increase in P and RNA content at low temperature provide high growth efficiency in cold environments^17,18^. Indeed, organisms with increased RNA content would be able to exert high growth rates during short periods of warm water exposure^4,19^. However, none of these studies explored the effect of regular changes in temperature as imposed for vertical migrating zooplankton in thermally stratified aquatic ecosystems.

The long term effect of temperature and food quality (C:P) on body P and the GRH was considered comparing populations from different systems^7^ or different clones in common gardens^20^. However, nutrient limitation was not considered under fluctuating temperature regimes. In aquatic ecosystems, organisms frequently move across temperature gradients in a regular pattern, as in zooplankton diel vertical migration (DVM)^21,22^. During DVM, zooplankton commonly descends through the thermocline to deep cold water, which reduces metabolic rates and hence secondary production^23–25^. Early experimental studies revealed that the growth of the cladoceran *Daphnia* in fluctuating temperatures decreased compared with those reared in warm water^25–27^. One consistent factor in all these experiments with varying temperature was that food was grown in non-nutrient limited media. The DVM was observed to be particularly significant in deep transparent oligotrophic or ultraoligotrophic systems^22,28,29^, in which low nutrient phytoplankton impose a nutrient limitation for zooplankton^30,31^. Thus, nutrient-limiting conditions for zooplankton should be included in the analysis of zooplankton growth under varying temperatures. In oligotrophic lakes, deep chlorophyll maxima (DCM) are common^32–34^, so food quantity increases at the upper layers of the hypolimnion^35^. In addition, food quality for grazer (phytoplankton C:P ratio) may not be even along the water column, with lower ratios at the hypolimnion^32,36^. Although DVM is the best known scenario of daily temperature change, it is not the only one, as shallow lakes may have daily temperature fluctuation of several degrees^37–39^. In addition, spatial temperature variation are also observed in shallow lakes, and diel horizontal migration (DHM) can also expose organisms to short-term fluctuating temperatures and food quality^8^. Thus, migrating organisms as during DVM and DHM, face several different gradients, such as temperature, food quantity and food quality. Regardless of the possible increase in food quality and quantity, DVM imposes regular temperature changes of several degrees that alone, may affect P, and RNA content, and growth rates. However, to what extent temperature fluctuation (in particular, short-term) in nutrient limited environments affects growth, P and RNA content in zooplankton, remains unclear.

North-Patagonian Andean lakes (40–42° S) are extremely transparent and have very low nutrient concentrations^33,40^. In particular, *D. commutata* growth rates were negatively affected when C:P was higher than 350^36^ and C:P over 550 limited their distribution^41^. The species displayed wide DVM (> 20 m) during which *D. commutata* experienced 10 °C of changing temperature (from 7 to 18 °C)^41,42^.

In this study, we aim to analyse the effect of fluctuating temperature under P limited conditions on the growth, P and RNA content of *Daphnia*. Hence, our objectives were to analyse if, under P-limiting conditions (high C:P), (i) *Daphnia* grown at low temperature will have higher P and RNA content compared to those at high temperature, (ii) when exposed to high temperature, *Daphnia* previously grown at low temperature will exhibit increased growth rates. And if in a P-limited system that experiences regular temperature changes (i.e. DVM), (iii) organisms will increase P, and thus RNA, content during the cold diurnal period and will grow during the warm night period, resulting in (iv) higher net growth than that expected by temperature alone. In this sense, we suggest that short-term fluctuating temperature alleviates nutrient limitation in *D. commutata*, via the increased P and RNA that can be used for compensatory growth during short periods of warm water exposure as suggested by Elser, et al.^4^. To test these predictions, we carried out a series of laboratory experiments in which growth of individuals was measured under different nutrient limiting conditions at low, medium, high and fluctuating temperature.

## Results

### Experiment 1

Growth rates, P and RNA content were contrasted between *D. commutata* grown at constant temperatures (10 °C or 20 °C) for 7 days with those grown at low temperature for 2 days and then translocated to warm temperature for the remaining 5 days. All neonates at the start of the experiment were of similar size (2.81 ± 0.15 µg ind^−1^) (ANOVA F_2,24_ = 3.14, *p* = 0.061). Neonates in the cold treatment reached, after 7 days, a mean size 5.66 µg ind^−1^, smaller than individuals of all other treatments. Mean size of individuals in the 20 °C treatment after 7 days was 21.58 µg ind^−1^. As expected, the size of individuals maintained at 10 °C for 2 days was equal to those of the cold treatment at the same age, but when translocated to 20 °C reached 17.62 µg ind^−1^ in the next 5 days. There were significant differences in size between all treatments with a significant interaction (two-way RM ANOVA time x treatment F_6,72_ = 54.51, *p* < 0.001). As all neonates were of similar size at the beginning, this interaction indicates that differences among treatments increased over time.

**Figure 1:**
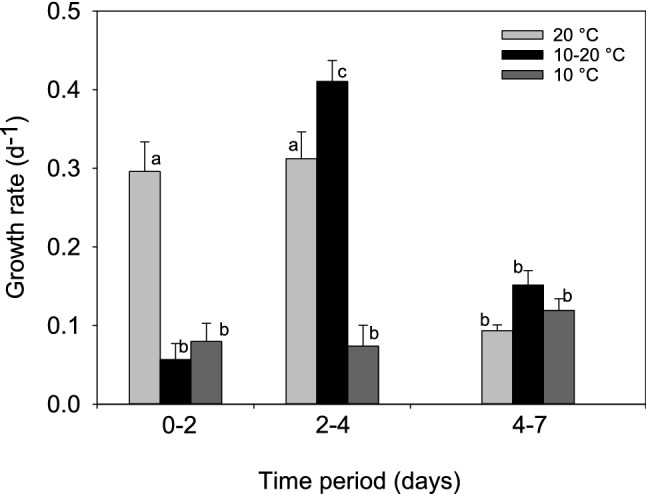
Growth rates of *D. commutata* in experiment 1. Error bars represent one standard error, and letters over the bars indicate homogeneous groups obtained with a Tukey test after a two-way ANOVA comparing among treatments and across time periods.

Growth rates also showed significant differences with a significant interaction (two-way RM ANOVA treatment F_2,24_ = 39.9, *p* < 0.001, time F_2,48_ = 25.40, *p* < 0.001, treatment x time F_4,48_ = 21.85, *p* < 0.001). During the 0–2 day interval the growth rate was higher for the warm treatment (0.29 d^−1^) than for the two treatments at 10 °C (cold and cold-warm, 0.079 and 0.056 d^−1^, respectively) (Fig. 1). However, the cold-warm treatment when translocated to 20 °C, had a higher growth rate (0.41 d^−1^ ± 0.026) (mean ± s.e.) for the interval 2–4 d, even higher than the warm treatment (0.31 d^−1^ ± 0.034) (Fig. 1) (for statistical significance see Fig. 1). The cold treatment remained with a low growth rate (0.073 d^−1^ ± 0.026). In the third interval (4–7 d), all treatments showed low growth rates (Fig. 1).

**Figure 2:**
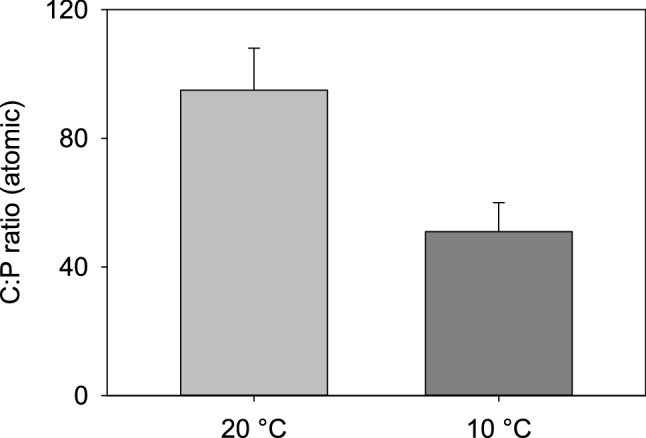
The C:P ratio of *D. commutata* after 2 days growth at 10 °C and 20 °C. Error bars represent one standard error.

**Figure 3:**
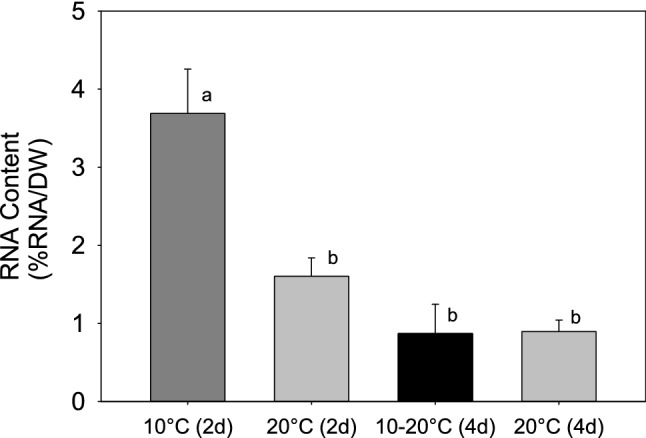
RNA content of *D. commutata*, in experiment 1. From left to right: After 2 days at 10 °C, after 2 days at 20 °C, at day 4 after spending 2 days at 10 °C and 2 days at 20 °C, and after 4 days at 20 °C. Error bars represent one standard error and letters over the bars indicate homogeneous groups.

After the first 2 days, we measured the C:P of *Daphnia* from the treatments at 20 °C (warm treatment) and at 10 °C (cold and cold-warm treatments). The results showed that the C:P of the individuals at 10 °C was significantly lower than at 20 °C (*t*-test *t*_(6)_ = 2.78, *p* = 0.031) (Fig. 2), indicating a higher P content in the individuals grown at 10 °C. Indeed, the P content (%P to DW) of individuals at 10 °C was 2.32%, while the P content of those at 20 °C was 1.2%. Individuals in the cold treatment had significantly higher RNA content after 2 days than individuals of the same age at 20 °C (3.6% and 1.6% respectively), and also higher than both treatments at day 4 (all at 20 °C) (one-way ANOVA F_3,28_ = 12.99, *p* < 0.001) (Fig. 3).

### Experiment 2

**Figure 4:**
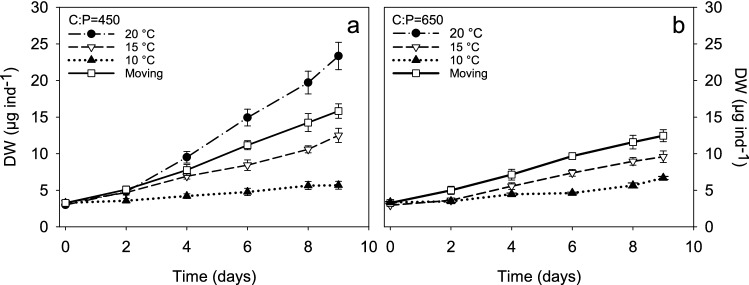
Size of *D. commutata* in (**a**) experiment 2 and (**b**) experiment 3. Error bars represent one standard error.

We compared four temperature treatments (cold, medium, warm and fluctuating) under P limiting condition (C:P = 450). As expected, organisms grew faster in warmer temperatures than in the cold treatment. Even though the mean temperature for both medium and fluctuating temperature treatments was very close (15 and 14.88 °C respectively), organisms in the fluctuating treatment grew faster than in the medium temperature, and even faster than the linear interpolation of the growth at 10 and 20 °C (Fig. 4a). There was a significant interaction between time and temperature (two-way RM ANOVA time x treatment F_15,250_ = 30.88, *p* < 0.0001), indicating that the difference in size between treatments increased with time. Differences in size between 15 °C and fluctuating treatment were detected on day 8 (a posteriori Bonferroni test, fluctuating vs 15 °C day 6, *p* = 0.094, day 8, *p* = 0.008).

RNA analysis in the fluctuating treatment (at 60 h, end of cold period 3.2%, and 72 h, end of warm period 3.9%), did not show significant differences (*t*-test *t*_(14)_ = 0.97, *p* = 0.347).

**Figure 5:**
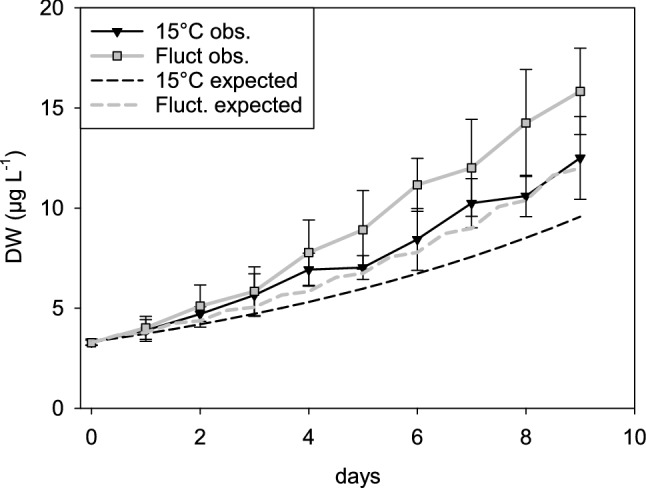
Observed and estimated sizes of *D. commutata* in the treatments 15 °C and fluctuating temperature. Solid lines and symbols: observed data, dashed lines and dash-dot-dot line estimated ones. Error bars of observed values represent 95% confidence intervals.

We calculated the expected sizes of the 15 °C and fluctuating treatments. The results obtained showed that the expected sizes were well below the observed ones (Fig. 5 solid versus dashed lines), with all expected values, except for the first two or three days, laying beyond the 95% confidence limits (Fig. 5). Noticeably, the expected and observed sizes of the fluctuating temperature were larger than the corresponding ones of the 15 °C treatment (Fig. 5 grey lines compared to black lines).

### Experiment 3

In this experiment we compared *D. commutata* growing at 15 °C with the ones in the fluctuating temperature, but with a higher P limitation than in experiment 2 (C:P = 650). We observed the same trend as in experiment 2, that is the individuals in the fluctuating temperature treatment grew more than at constant 15 °C (Fig. 4b). As expected, animals in experiment 3 fed with lower food quality, grew less than those of experiment 2. However, the decrease in size of *Daphnia* in experiment 3 (lower food quality, C:P = 650) compared to experiment 2 (C:P = 450), was smaller in the fluctuating treatment (21%) compared to the constant 15 °C (26%), and these differences were significant (two way ANOVA Temp F_1,36_, *p* = 0.005; C:P F_1,36_ *p* = 0.004), and in the a posteriori Tukey test, all comparisons were significant but C:P within fluctuating (*p* = 0.065).

## Discussion

In this study we show that under fluctuating temperature regimes, as those imposed to migrating zooplankton, organisms can growth more than predicted by temperature alone. In our experiments, in the fluctuating temperature regime, organisms reached sizes not only larger than expected by the Q_10_, but even larger than the mean size between the 10 °C and 20 °C treatments, assuming a linear interpolation as a conservative supposition. However, metabolic functions including growth do not increase linearly with temperature^1,43^. According to the exponential response of metabolic and growth rates to temperature and the Q_10_ estimations, it would be expected that the growth at mean temperature (15 °C) should be lower than the linear interpolation of two temperatures (10 and 20 °C). In addition, the sizes of the 15 °C treatment were also larger than expected by Q_10_. These larger than expected sizes, would imply that other factor than temperature, is also affecting growth.

Our experiments also showed that *D. commutata* grown at low temperature had significantly more P and RNA content than individuals grown at higher temperature. In addition, individuals raised at low temperature (10 °C) also responded to increasing temperature (20 °C) with increased growth. Woods, et al.^16^ found that there is a general trend of P and RNA increase at low temperature, probably because more rRNA is needed to maintain synthesis at low temperatures^18^. The efficiency of synthesis, the synthesis per rRNA unit, decreases with the decrease in temperature so an increase in rRNA, and hence in P, may compensate at least in part for the decrease in synthesis^17^. Our results on RNA content gave rather low values of this compound, however, low RNA content were also obtained in experiments with *Daphnia galeata* and *D. pulicharia* under similar P-limited conditions (C:P of food 457)^44^. In our first experiment we observed that growth rate and RNA content showed opposite patterns. High RNA content was registered when GR was low (0–2 days at 10 °C) while high GR was observed with low RNA content (0–2 days at 20 °C and 2–4 days in the 10–20 °C treatment). A decoupling of the RNA-GR relationship was observed when the GRH was tested across temperatures, and was attributed to a less RNA requirement at high temperatures due to faster synthesis^7^. In our first experiment, during the first two days, GR in the 10 °C treatment was low, and in the next period when translocated to 20 °C (2–4 days) it was high. The GR is calculated for a certain period of time, while the RNA is a measurement of a specific moment, that does not integrate the period as GR does. Consequently, the GR of the 2–4 period does not depend on the RNA at the end of the period, but at the beginning of it, which was high. As growth takes place, the increase in size produces a dilution effect if DW increases more than P uptake.

Whether P limitation increases or decreases with temperature is still under discussion^15^. In any case, individuals grown at lower temperature have a higher P content, and this condition is achieved in 48 h, showing that is not necessarily an adaptive condition of populations living permanently at different temperatures^4^. It can be assumed that individuals at low temperature would incorporate P from the diet at a similar rate than individuals at higher temperature, but their growth will be lower, so the P and RNA content is not diluted with the increase in body mass. Alternatively, at 20 °C the body grows faster, so the P and RNA content (µg RNA µg DW^−1^) is lower because of a dilution effect with higher DW. Indeed, in our first experiment, after 48 h individuals grown at 10 °C and 20 °C had similar P and RNA per individual, but very different P and RNA content due to the larger size of individuals grown at 20 °C. Consequently, when individuals growing at 10 °C for 2 days were transferred to 20 °C, the higher RNA content (%RNA), allowed them to increase their growth rate reaching even higher growth rates than individuals growing permanently at 20 °C in the 0–2 days. That is, when individuals at 10 °C were changed to 20 °C, they were less P limited and the combined effect of high temperature and high P and RNA content, allowed a faster growth.

Although the activity of alkaline phosphatase enzyme (APA) has been considered as a proxy of P limitation, it has been shown that at low temperatures, APA increases to compensate for the lower enzymatic efficiency^10^. These measurements were carried out after 6 days of food and temperature acclimation, but we don’t have information on how quick APA can change. Some defense enzymes, as those required to neutralize reactive oxygen species that induce oxidative stress, can react very quickly (1 h)^45^, while APA changes were observed after 4 h of induced oxidative stress (ultraviolet light or H_2_O_2_ exposure)^46^. Because of its function, that is very different from that of antioxidant enzymes, it is unlikely that APA would respond so fast. This means that during regular short time temperature fluctuation as in DVM (mimicked in our experiment) APA would be not as high as the low temperature would induce, but much higher than that of 20 °C. If APA does not respond quickly, when individuals migrate upwards during dusk, they will be at higher temperature (fast growing) with much more APA than individuals permanently at 20 °C, so they could uptake P more efficiently. Probably, this condition of high APA can be extended for few hours, giving the organism a better somatic condition for growth. Then, they return to colder waters, and the growth its reduced by the low temperature, but some hours later, APA would be again increased. This effect would explain the higher growth rate observed in the fluctuating temperature treatment (higher than expected only by temperature) and also the lack of differences in P and RNA content in the different phases of the temperature cycle.

The increased growth that occurs following a period of restriction (mainly food) was described as compensatory growth^47,48^. However, copepods that were fed with P-deficient food did not show this response in growth^49^. In our experiment we did not vary food P content or food quantity, but temperature, and we did observe an increased growth when the temperature was risen to 20 °C. This “thermal compensatory” growth lasts for a short time, probably while %RNA remains high, but as the individuals grow quickly, the %RNA decreases along with the increase in size, as seen in days 2–4 and 4–7 of experiment 1.

In the fluctuating temperature treatments, we expected that at the end of the 12 h cold period, *D. commutata* would have more RNA content than at the end of the warm period (12 h later), similar to the results obtained in experiment 1 after 48 h. However, in the fluctuating temperature regime, the RNA content was similar to the cold treatment of experiment 1, so what we did not see was a significant decrease during the warm period, remaining high during both phases of the temperature cycle. In addition to the temperature effect on APA, discussed previously, it could be assumed that the cold period is like a nutrient pulse in the sense of the Droop model^50^, so the increase in P content in *D. commutata* would have occurred in a very short time period, as it occurs in algae when a P-spike is added^18,51,52^. However, during the cold period P content would increase much more slowly than expected by the Droop model with a P pulse. Phosphorus uptake may not change during the cold periods, but because of the slow growth, relative P content increases. The result is that under this fluctuating regime, individuals spending the 20 °C period with the RNA content of the 10 °C period can grow faster than expected by temperature alone.

Daily migration to cold water was seen as a behaviour that lowers growth, but with the advantage of avoiding visual predation^23,39^ or damaging wavelengths^22^. However, here we show that under nutrient-limitation, daily migration can have other advantages. The time spent in the cold water will be in part compensated by increased growth while in warm water, due to the P uptake during the cold period. It has been argued that during DVM, the organisms fast while in the cold deep water, since the food was only in the warm illuminated epilimnion^21,27^. However, the presence of a deep Chlorophyll maxima is frequent in many lakes around the world^53–55^. Moreover, the depth of the deep chlorophyll maxima depends primarily on light more than on other factors^33,35^, and in transparent systems, this deep maxima lays below the thermocline, so cold water layers are not food depleted. In addition, the C:P ratio of the algae would be lower (better food quality for grazers) at the hypolimnion^32,36^ where the DCM lays. This implies that migrating individuals not only do not face fasting while in the deep cold water as previously suggested^27^, but may have more and better food. In our experiments, we did not vary food quantity or quality during changes in temperature (simulating DVM), however, we showed that fluctuating temperature alone increases growth. In addition, the effect of increased food quality in deep water^32,36^ would imply that grazers would have more P and the effect we reported here would be enhanced. Under a DVM regime, the temperature alternation with the concomitant nutrient acquisition in cold water, would allow migrating organisms to alleviate the effect of severe nutrient limitation maintaining population growth. Thus, the costs of vertical migration, in terms of reduced growth rate^27^, may be much lower than determined in early experiments in which *Daphnia* were fed with P-replete algal food. Global warming is affecting the temperature structure of lakes, increasing the stratification period, but also increasing the thermal difference between epilimnion and hypolimnion^56,57^. These changes in lake temperature will interact with nutrient limitation for consumers, but, according to our results, organisms may be able to face these changes if they can still regularly move from a cold hypolimnion to a warmer epilimnion.

## Methods

All experiments utilised a clone culture of *Daphnia commutata* originally started from a female collected from Lake Mascardi (41°15′–41°25′S; 71°28′–71°39′ W) in Patagonia, Argentina, and maintained in the laboratory for more than ten years in P-free COMBO medium modified from Kilham, et al.^58^, fed *Chlamydomonas reinhardtii* in a chamber at 15 °C. The *C. reinhardtii* culture was grown in MBL medium^59^. The P source (K_2_HPO_4_) of the algal culture was controlled in each experiment as detailed below in the experimental design. All experiments were conducted in water baths in growth chambers at the selected temperature and subject to a photoperiod of 12:12 (light:dark) with dim light (4 µmol photon m^−2^ s^−1^). We used this light level for the illuminated phase of the experimental day cycle because during the day *D. commutata* migrating individuals are located in the hypolimnetic deep cold water, around the 1% surface irradiance^41,42^. All experiments began using neonates (< 12 h old) from adult females fed with algae grown in P-limited media according to each experiment.

### Experimental design

#### Experiment 1

Neonate (< 12 h old) *D. commutata* were placed individually in 15 mL tubes with P-free COMBO and P-limited but not quantity limited food (*Chamydomonas reinhardtii*: 1 mg C L^−1^, C:P atomic ratio of 450). Previous experiments showed that at a C:P of 450 *D. commutata* were P limited and it is a food C:P ratio commonly encountered by *D. commutata* in its natural environment^36,41,60^. Medium and food were replaced every day by pipetting the individual to a clean tube with fresh medium and food. Adults of this species (2 mm in body length) have a clearance rate of 4 mL d^−1^^61^. Assuming a clearance rate of 4 mL d^−1^, much higher than for juveniles, at the end of the 24 h period, the C concentration would have reduced to 0.75 mg C L^−1^, thus, in our experiments, neonates and juveniles, that reached 1.6 mm in body length, were not food limited between food replacements. The treatments were (a) cold (b) cold-warm and (c) warm (Fig. S1). Briefly, 80 individuals were placed at 10 °C and 65 individuals at 20 °C. After two days, individuals from the cold treatment (10 °C) were randomly distributed as follows: 15 were kept at 10 °C for cold water growth estimations, 25 were switched to 20 °C (cold-warm treatment), 30 used for C and P determinations (3 replicates of 5 individuals for P and equal set for C) and 10 for RNA determination. On the same day, of the 65 individuals at 20 °C (warm treatment), 30 were designated for C and P content, and 10 for RNA content. On day 4, 10 individuals from the cold-warm and warm treatments were designated for RNA determination and the remaining 15 each of cold-warm and warm treatments were kept at 20 °C for growth estimations until the end of the experiment. Growth estimations were based on size measurements of each individual in each treatment at the initial time (0 day), 2 days, 4 days and 7 days as detailed in the measurements section.

#### Experiment 2

*D. commutata* was subjected to four treatments (10 °C, 15 °C, 20 °C and fluctuating temperature regime). In all treatments, individuals were placed individually in 15 mL tubes, with P-free COMBO and food (*C. reinhardtii* 1 mg C L^−1^) with a C:P atomic ratio of 450. Medium and food were replaced every day by pipetting the individual to a clean tube with fresh medium and food. The treatments were (a) cold: 15 individuals were placed at 10 °C for 9 days; (b) medium: 15 individuals were placed at 15 °C for 9 days; (c) warm: 15 individuals were placed at 20 °C for 9 days; and (d) fluctuating regime: 35 individuals were placed for 9 days in a system that changed the temperature in a continuous cycle of 12 h at 9 °C and 12 h at 20 °C, with a dim light (4 µmol photon m^−2^ s^−1^) during the cold period and darkness during the warm period. This treatment was started with 35 individuals, and at 60 h (end of a cold period) and 72 h (end of a warm period), 10 individuals were sampled for RNA measurement. Thus, 15 individuals were used for size and growth, and 20 for RNA analysis. The rate of change between the two temperatures was 0.14 °C min^−1^, requiring 75 min to reach the new temperature (Fig. S2). Sharp changes in temperature exert a stress effect^24^. In fact, Mikulski, et al.^24^ found that growth rates and heat shock proteins dropped when the temperature changed at rates higher than ~ 0.4 °C min^−1^. However, in our experiments, the rate of temperature change (0.14 °C min^−1^) was much lower, allowing us to assume no stress was experienced at this temperature change rate.

The temperature was monitored with a data logger (HOBO Pendant Temp UA-001-08) that allowed for determination of possible temperature cycle anomalies during the experiment. The mean temperature of the water bath for the nine days of the experiment was 14.88 °C. This treatment was designed to mimic the temperature change during DVM, assuming equal time spent in epilimnion and hypolimnion. Individuals from all treatments were photographed every day. At the end of the warm period of the fluctuating treatment, the media and food were replaced.

#### Experiment 3

Similar to experiment 2, we ran a third experiment with 2 treatments (15 °C and fluctuating temperature) with identical conditions but with a higher P limitation (C:P = 650). This condition of high C:P ratio was observed to be limiting for *D. commutata* growth^36^, and distribution^41^. In this case, we used 15 individuals for each treatment, as only size was measured in this experiment. This experiment was carried out to detect if the fluctuating temperature had similar effects compared with the mean temperature when individuals were more P limited.

As expected for this species in these food qualities, in all the three experiments *D. commutata* females did not develop eggs during the growth phase studied (9 days).

### Size and growth estimations

Size and growth were estimated according to^44^ using lateral images that allow measurement of individuals while the experiment is ongoing. Lateral images were obtained at 20 × magnification (Olympus SZX9 microscope) with a digital camera (5Mp). Images were analysed with ImageJ software (ImageJ, ver. 1.52a, NIH, USA). Biomass was estimated from area-dry weight regressions estimated for this species grown under similar food qualities^60^. Growth rates were estimated for each interval (0–2, 2–4 and 4–7) of experiment 1 and daily for experiments 2 and 3 as $GR=\frac{\mathrm{ln}{DW}_{f}-\mathrm{ln}{DW}_{i}}{t}$ in which *DW*_*f*_ and *DW*_*i*_ are the dry weights at the end and the beginning of the growth interval, respectively, and *t* is the time in days between measurements. Growth rates of experiment 2 were used for calculation of the Q_10_ (see “Expected sizes and growth rates” section).

Carbon concentration of the food (all experiments) and of *D. commutata* from experiment 1 was estimated as follows. Algae: 3 mL of culture were filtered on GF/F precombusted filters and analysed in a Thermo Finnigan EA1112 elemental analyser. For *D. commutata*, 5 individuals were pooled on a GF/F precombusted filter and analysed as the algae. Phosphorus was analysed on equal volume of algae culture or *Daphnia* number in MilliQ water and combusted with Sodium persulfate at 125 °C and analysed with the ascorbate-reduced molybdenum method^62^.

Methods in Gorokhova and Kyle^63^ and Hessen, et al.^18^ were followed for RNA analyses: individuals were collected with forceps and gently rinsed in MilliQ RNAse-free water, placed alive individually in nuclease-free Eppendorf tubes, immediately snap-frozen in liquid N and then stored at − 80 °C until RNA analysis. Before the analysis, 100 µL of sarcosyl 1% in TE buffer (Tris–EDTA buffer, pH 7.5) was added to the tube, the tube kept on ice and sonicated for 90 s (Labsonic M, Sartorius) then diluted (1:5) with TE buffer. The tubes were then centrifuged (12,000 RPM at 4 °C) and the supernatant was used for analysis. For each individual 100 µL extracted supernatant was placed in a 96-well microplate and a second 100 µL in a contiguous well. To the latter, we added 1 µL of RNAse and incubated the plate in the dark at 37 °C for 1 h. Finally, 100 µL of Ribogreen was added to each well and the fluorescence read in a plate reader (Synergy HTX, BioTek) with excitation at 480 nm and emission at 520 nm. A calibration curve was placed in the first two lines of the plate, and the estimation was carried out as the difference in fluorescence between untreated and RNAse-treated wells. RNA content was expressed as percent of the RNA on dry weight (RNA/DW*100).

### Expected sizes and growth rates

We estimated the expected sizes at 15 °C (${\widehat{S}}_{\left(15\right)t}$) based on the estimation of Q_10_, calculated on 10 °C and 20 °C treatments of experiment 2, Q_10_ was calculated as

$${Q}_{10}={\left(\frac{{GR}_{2}}{{GR}_{1}}\right)}^{\left(\frac{10}{{T}_{2}-{T}_{1}}\right)}$$

where GR_1_ and GR_2_ are growth rates at temperatures T_1_ and T_2_ (T_2_ > T_1_).

Based on the Q_10_ and the GR_10_ we estimated the expected growth rates at 15 °C (${\widehat{GR}}_{15}$) as

$${\widehat{GR}}_{15}=G{R}_{10}\times {\left({Q}_{10}\right)}^{\frac{(15-10)}{10}}$$

Then, we estimated the expected sizes at 15 °C (${\widehat{S}}_{\left(15\right)t}$) as

$${\widehat{S}}_{\left(15\right)t}={S}_{0}\times {e}^{\left({\widehat{GR}}_{15}\times t\right)}$$

We also estimated the expected sizes of the fluctuating temperature treatment (${\widehat{S}}_{\left(f\right)t}$) as successive cycles of alternating half day growing at a rate GR_10_ (10 °C) and half at a rate GR_20_ (20 °C). Although the daily growth rates (between consecutive days) were very similar to the mean growth rate (0–9 days), in this case we used for each day the observed GR for that period.

### Statistical analyses

Comparison of sizes at the beginning of the three experiments was conducted with a one-way ANOVA. Size change with time in each experiment and growth rates of experiment 1 were analysed with a two-way Repeated Measures ANOVA. Comparisons of C:P in experiment 1, and RNA content in experiment 2 were carried out with a *t*-test. For analysis of RNA content in experiment 1, we applied a one-way ANOVA. In all ANOVA analyses with significant results, a posteriori Tukey (for one-way ANOVA) and Bonferroni (for RM ANOVA) tests were applied. In all cases, normality and homoscedasticity were verified before the analysis.

## Supplementary Information

Supplementary Information.

## Data Availability

All data are available at the Universidad Nacional del Comahue data repository http://rdi.uncoma.edu.ar//handle/123456789/16222.

## References

[1] Brown JH, Gillooly JF, Allen AP, Savage VM, West GB (2004). Toward a metabolic theory of ecology. Ecology.

[2] Dillon ME, Wang G, Huey RB (2010). Global metabolic impacts of recent climate warming. Nature.

[3] Elser JJ (2000). Biological stoichiometry from genes to ecosystems. Ecol. Lett..

[4] Elser J, Obrien W, Dobberfuhl D, Dowling T (2000). The evolution of ecosystem processes: growth rate and elemental stoichiometry of a key herbivore in temperate and arctic habitats. J. Evol. Biol..

[5] Hessen DO, Elser JJ, Sterner RW, Urabe J (2013). Ecological stoichiometry: An elementary approach using basic principles. Limnol. Oceanogr..

[6] Hessen DO, Faerovig PJ, Andersen T (2002). Light, nutrients, and P : C ratios in algae: Grazer performance related to food quality and quantity. Ecology.

[7] Moody EK, Rugenski AT, Sabo JL, Turner BL, Elser JJ (2017). Does the growth rate hypothesis apply across temperatures? Variation in the growth rate and body phosphorus of neotropical benthic grazers. Front. Environ. Sci..

[8] Prater C, Wagner ND, Frost PC (2018). Seasonal effects of food quality and temperature on body stoichiometry, biochemistry, and biomass production in *Daphnia* populations. Limnol. Oceanogr..

[9] Boersma M (2016). Temperature driven changes in the diet preference of omnivorous copepods: No more meat when it's hot?. Ecol. Lett..

[10] Wojewodzic MW, Kyle M, Elser JJ, Hessen DO, Andersen T (2011). Joint effect of phosphorus limitation and temperature on alkaline phosphatase activity and somatic growth in Daphnia magna. Oecologia.

[11] Starke CWE, Jones CLC, Burr WS, Frost PC (2020). Interactive effects of water temperature and stoichiometric food quality on *Daphnia pulicaria*. Freshwat. Biol..

[12] Ruiz T (2020). U-shaped response Unifies views on temperature dependency of stoichiometric requirements. Ecol. Lett..

[13] Persson J, Wojewodzic MW, Hessen DO, Andersen T (2011). Increased risk of phosphorus limitation at higher temperatures for *Daphnia magna*. Oecologia.

[14] Malzahn AM, Doerfler D, Boersma M (2016). Junk food gets healthier when it's warm. Limnol. Oceanogr..

[15] Cross WF, Hood JM, Benstead JP, Huryn AD, Nelson D (2015). Interactions between temperature and nutrients across levels of ecological organization. Glob. Change Biol..

[16] Woods HA (2003). Temperature and the chemical composition of poikilothermic organisms. Funct. Ecol..

[17] Cotner JB, Makino W, Biddanda BA (2006). Temperature affects stoichiometry and biochemical composition of *Escherichia coli*. Microb. Ecol..

[18] Hessen DO (2017). Changes in stoichiometry, cellular RNA, and alkaline phosphatase activity of *Chlamydomonas* in response to temperature and nutrients. Front. Microbiol..

[19] Van Geest GJ, Sachse R, Brehm M, van Donk E, Hessen D (2010). Maximizing growth rate at low temperatures: RNA:DNA allocation strategies and life history traits of Arctic and temperate Daphnia. Polar Biol..

[20] Prater C, Wagner ND, Frost PC (2017). Interactive effects of genotype and food quality on consumer growth rate and elemental content. Ecology.

[21] Lampert W (1989). The adaptive significance of diel vertical migration of zooplankton. Funct. Ecol..

[22] Williamson CE, Fischer JM, Bollens SM, Overholt EP, Breckenridge JK (2011). Towards a more comprehensive theory of zooplankton diel vertical migration: Integrating ultraviolet radiation and water transparency into the biotic paradigm. Limnol. Oceanogr..

[23] Dawidowicz P, Loose CJ (1992). Metabolic costs during predator-induced diel vertical migration of *Daphnia*. Limnol. Oceanogr..

[24] Mikulski A, Grzesiuk M, Rakowska A, Bernatowicz P, Pijanowska J (2017). Thermal shock in *Daphnia*: cost of diel vertical migrations or inhabiting thermally-unstable waterbodies?. Fund. Appl. Limnol..

[25] Reichwaldt ES, Wolf ID, Stibor H (2005). Effects of a fluctuating temperature regime experienced by *Daphnia* during diel vertical migration on *Daphnia* life history parameters. Hydrobiologia.

[26] Orcutt JD, Porter KG (1983). Diel vertical migration in zooplankton. Constant and fluctuating temperature effects on life history parameters of Daphnia. Limnol. Oceanogr..

[27] Stich HB, Lampert W (1984). Growth and reproduction of migrating and non-migrating *Daphnia* species under simulated food and temperature conditions of diurnal vertical migration. Oecologia.

[28] Fischer JM (2015). Diel vertical migration of copepods in mountain lakes: The changing role of ultraviolet radiation across a transparency gradient. Limnol. Oceanogr..

[29] Kessler K, Lockwood RS, Williamson CE, Saros JE (2008). Vertical distribution of zooplankton in subalpine and alpine lakes: Ultraviolet radiation, fish predation, and the transparency-gradient hypothesis. Limnol. Oceanogr..

[30] Bergström A-K, Karlsson J, Karlsson D, Vrede T (2018). Contrasting plankton stoichiometry and nutrient regeneration in northern arctic and boreal lakes. Aquat. Sci..

[31] Sterner RW (2008). On the phosphorus limitation paradigm for lakes. Int. Rev. Hydrobiol..

[32] Sterner RW (2011). C: N: P stoichiometry in Lake superior: Freshwater sea as end member. Inland Waters.

[33] Modenutti BE (2013). Environmental changes affecting light climate in oligotrophic mountain lakes: The deep chlorophyll maxima as a sensitive variable. Aquat. Sci..

[34] Longhi ML, Beisner BE (2009). Environmental factors controlling the vertical distribution of phytoplankton in lakes. J. Plankton Res..

[35] Leach TH (2018). Patterns and drivers of deep chlorophyll maxima structure in 100 lakes: The relative importance of light and thermal stratification. Limnol. Oceanogr..

[36] Laspoumaderes C (2013). Glacier melting and stoichiometric implications for lake community structure: Zooplankton species distributions across a natural light gradient. Glob. Change Biol..

[37] Jacobs AFG, Jetten TH, Lucassen D, Heusinkveld BG, Joost PN (1997). Diurnal temperature fluctuations in a natural shallow water body. Agric. For. Meteorol..

[38] Vilas MP, Marti CL, Adams MP, Oldham CE, Hipsey MR (2017). Invasive macrophytes control the spatial and temporal patterns of temperature and dissolved oxygen in a shallow lake: A proposed feedback mechanism of macrophyte loss. Front. Plant Sci..

[39] Burks RL, Lodge DM, Jeppesen E, Lauridsen TL (2002). Diel horizontal migration of zooplankton: Costs and benefits of inhabiting the littoral. Freshwat. Biol..

[40] Morris DP (1995). The attenuation of solar UV radiation in lakes and the role of dissolved organic carbon. Limnol. Oceanogr..

[41] Balseiro EG, Modenutti BE, Queimaliños C, Reissig M (2007). *Daphnia* distribution in Andean Patagonian lakes: Effect of low food quality and fish predation. Aquat. Ecol..

[42] Modenutti BE, Wolinski L, Souza MS, Balseiro EG (2018). When eating a prey is risky: Implications for predator diel vertical migration. Limnol. Oceanogr..

[43] Gillooly JF, Charnov EL, West GB, Savage VM, Brown JH (2002). Effects of size and temperature on developmental time. Nature.

[44] Acharya K, Kyle M, Elser JJ (2004). Biological stoichiometry of *Daphnia* growth: An ecophysiological test of the growth rate hypothesis. Limnol. Oceanogr..

[45] Souza MS, Hansson L-A, Hylander S, Modenutti BE, Balseiro EG (2012). Rapid enzymatic response to compensate UV radiation in copepods. PLoS ONE.

[46] Wolinski L, Modenutti B, Souza MS, Balseiro E (2016). Interactive effects of temperature, ultraviolet radiation and food quality on zooplankton alkaline phosphatase activity. Environ. Pollut..

[47] Xie J (2015). Physiological effects of compensatory growth during the larval stage of the ladybird Cryptolaemus montrouzieri. J. Insect Physiol..

[48] Dmitriew C, Rowe L (2005). Resource limitation, predation risk and compensatory growth in a damselfly. Oecologia.

[49] Malzahn AM, Boersma M (2012). Effects of poor food quality on copepod growth are dose dependent and non-reversible. Oikos.

[50] Droop MR (1973). Some thoughts on nutrient limitation in algae. J. PhycoI..

[51] Boersma M (2000). The nutritional quality of P-limited algae for *Daphnia*. Limnol. Oceanogr..

[52] Plath K, Boersma M (2001). Mineral limitation of zooplankton: Stoichiometric constraints and optimal foraging. Ecology.

[53] Barbiero RP, Tuchman ML (2001). Results from the US EPA's biological open water surveillance program of the Laurentian Great Lakes: II. Deep chlorophyll maxima. J. Great Lakes Res..

[54] Camacho A (2006). On the occurrence and ecological features of deep chlorophyll maxima (DCM) in Spanish stratified lakes. Limnetica.

[55] Pérez GL, Queimaliños CP, Modenutti BE (2002). Light climate and plankton in the deep chlorophyll maxima in North Patagonian Andean lakes. J. Plankton Res..

[56] Magee MR, Wu CH (2017). Response of water temperatures and stratification to changing climate in three lakes with different morphometry. Hydrol. Earth Syst. Sci..

[57] Niedrist GH, Psenner R, Sommaruga R (2018). Climate warming increases vertical and seasonal water temperature differences and inter-annual variability in a mountain lake. Clim. Change.

[58] Kilham SS, Kreeger DA, Lynn SG, Goulden CE, Herrera L (1998). COMBO - A defined freshwater culture medium for algae and zooplankton. Hydrobiologia.

[59] Guillard RRL, Lorenzen CJ (1972). Yellow-green algae with chlorophyllide c. J. Phycol..

[60] Balseiro EG, Souza MS, Modenutti BE, Reissig M (2008). Living in transparent lakes: Low food P: C ratio decreases antioxidant response to ultraviolet radiation in* Daphni*a. Limnol. Oceanogr..

[61] Laspoumaderes C, Souza MS, Modenutti BE, Balseiro E (2017). Glacier melting and response of *Daphnia* oxidative stress. J. Plankton Res..

[62] APHA. *Standard methods for the examination of water and wastewater*. (American Public Health Association, AWWA, 2005).

[63] Gorokhova E, Kyle M (2002). Analysis of nucleic acids in *Daphnia*: development of methods and ontogenetic variations in RNA-DNA content. J. Plankton Res..

