# Supplementary material for: Short term fluctuating temperature alleviates Daphnia stoichiometric constraints
Source: Sci Rep. 2021 Jun 11;11:12383. doi: 10.1038/s41598-021-91959-w (PMC8196208; doi:10.1038/s41598-021-91959-w)

Short term fluctuating temperature alleviates *Daphnia* stoichiometric constraints.

Esteban Balseiro, Cecilia Laspoumaderes, Facundo Smufer, Laura Wolinski, Beatriz Modenutti.

Supplementary material

Figure S1. Experimental design: A. Temperature regime of experiment 1, with indications of the moments where size, C:P ratio and RNA content were measured. B. Temperature fluctuating regime of experiments 2 and 3

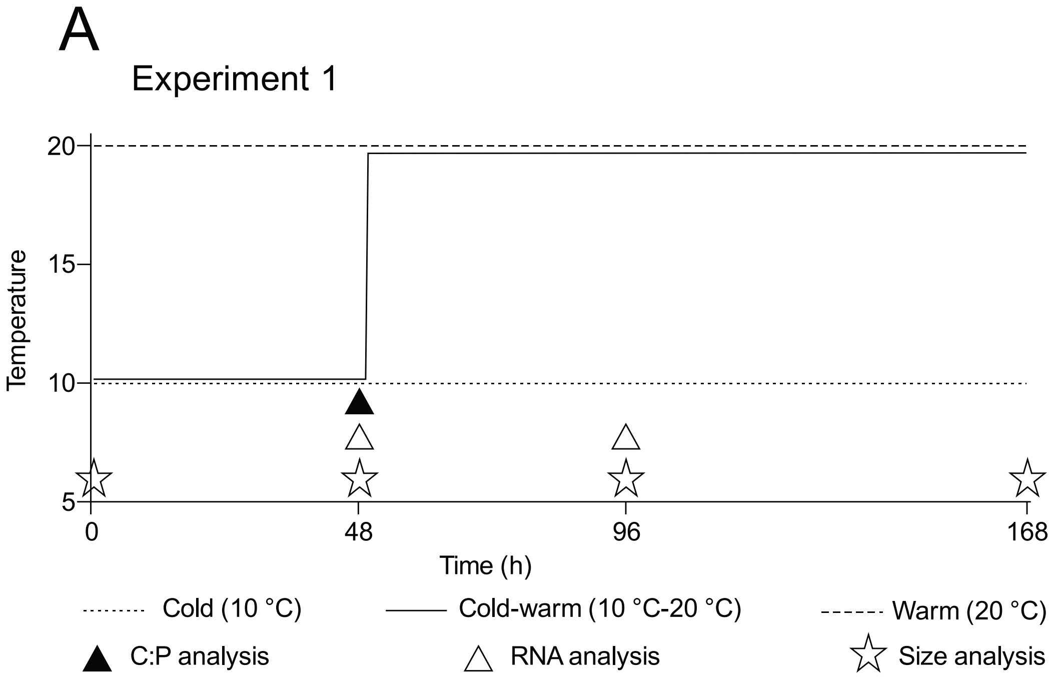

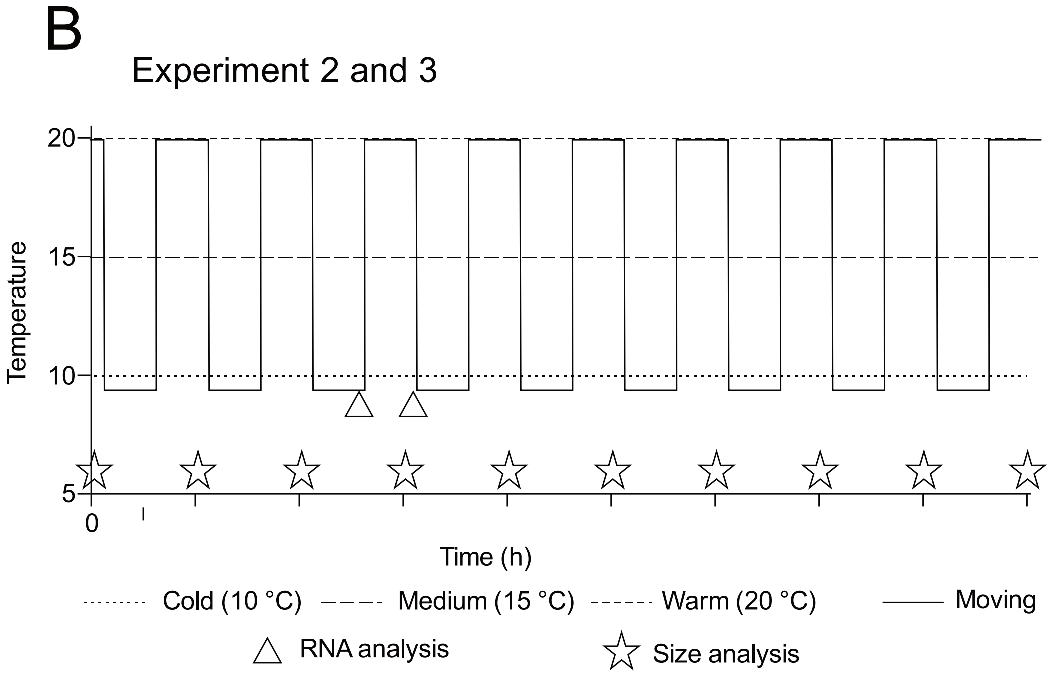

Figure S2. Detail of the temperature regime of the fluctuating temperature treatment of Experiments 2 and 3, shaded areas indicate dark period. During day the light intensity was set at 4 µmol photon m^{-2} s^{-1}, mimicking dim light at deep hypolimnetic layers.

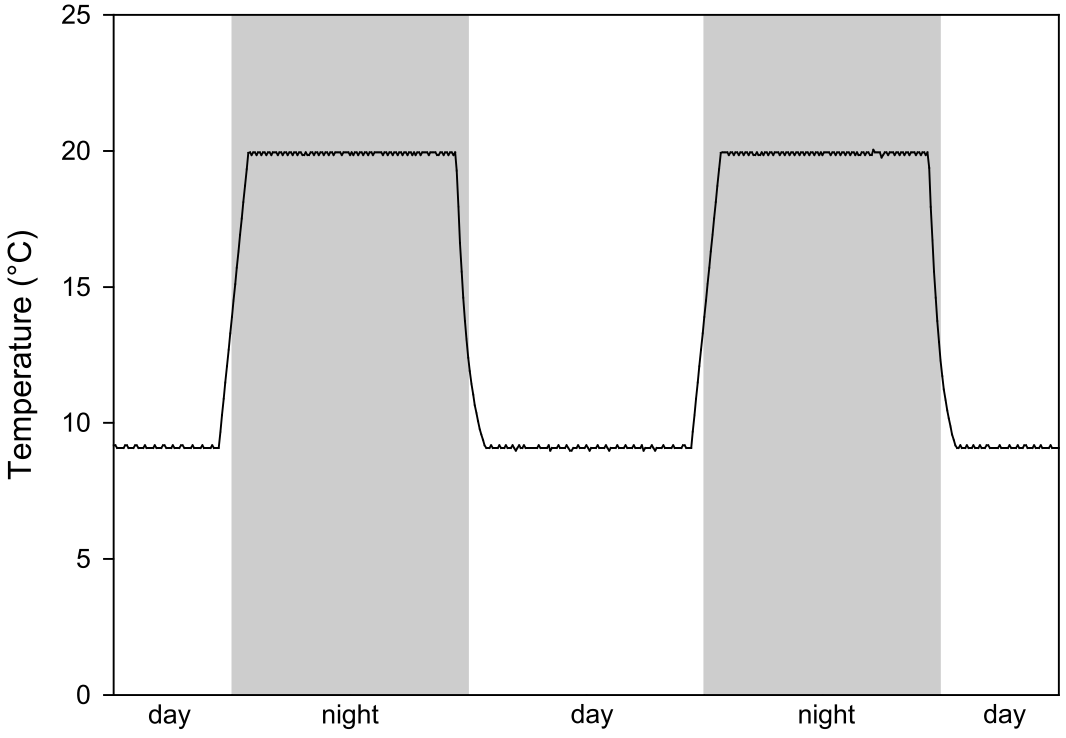

Supplement: Supplementary file 1 — Supplementary Information. [file 41598_2021_91959_MOESM1_ESM.pdf]
